# Ethics of Medical Archival Internet Research Data

**DOI:** 10.2196/43754

**Published:** 2023-01-31

**Authors:** Tsuriel Rashi, Elad Yom-Tov

**Affiliations:** 1 School of Communication Ariel University Ariel Israel; 2 Microsoft Herzliya Israel

**Keywords:** ethic, archival internet research data, three laws of robotics

## Abstract

Medical research based on internet archive data, which in some ways is quite different from other data-based studies, is becoming more and more common. Despite its uniqueness and the challenges that characterize it, clear ethical rules designed to guide practitioners in this field have not yet been written. This article points to the lacuna that exists in legal and ethical texts today and offers an ethically balancing alternative. Among other features, the balance is based on the famous three laws of robotics by Asimov and a series of values, including transparency, accountability, fairness, and privacy.

## Introduction

### The Status of Medical Archival Internet Research

Internet services collect large quantities of information generated through the interactions of users with its services [1]. These data (referred to henceforth as “internet data”) are important for improving these services. They provide a better experience for users, and they have been shown to be of value for both individual and public health [2]. For example, search engine queries have been applied to medical applications as a secondary use, demonstrating that they can be used to screen people for a range of medical conditions, including several types of cancer [3], Parkinson disease [4], and stroke [5], among others. The services that collect these data are not necessarily subject to institutional review boards, so there are no clear ethical guidelines or government policy governing their use for research (both medical and otherwise) [6]. Some researchers have suggested maintaining the status quo [7], others have recommended adopting “best practices,” [8] whereas a third camp is advocating measures that would effectively ban research that uses internet data [9].

In recent years, studies have been published that have dealt with the ethics of medical research. The 4 fundamental ethical principles that are being underscored are autonomy, nonmaleficence, beneficence, and justice [10]. There have also been studies published that deal with the ethics of internet research, which cite a number of principles concerned with security for the individual, anonymity, safety, and use of mined data and information gathered by either passive or active means, among others [11]. McKee and Porter [12] point to key ethical issues involved in conducting archival research. Based on an examination of cases and interviews with leading archival researchers, they discuss several ethical questions and offer a heuristic guide to ethical decision-making. Yet none of the above-mentioned studies combines the different types of medical research information gathering and examines their uniqueness, so they do not relate to the need for a clear ethical statement regarding the use of medical research–based data acquired from the public.

Medical research based on archival internet data differs from studies using archival data from more traditional sources in several important ways. Below, we discuss these differences as they pertain to the usability of the data and to the ethical implications of employing them. We note that, when referring to internet data, we allude to any data collected by an internet service provider pertaining to the activity of humans, including, for example, search queries and technical parameters of the interaction between users and the search engine (eg, number of clicked links), social media postings, or phone location data.

The first and most obvious difference between internet and traditional data sources is in the control over the data collected. Traditional data are often collected under the full control of the investigator, following a carefully monitored intervention. Internet data are collected for the two following main reasons, neither of which is research: to provide information to users and to facilitate improving the service that provides them [13]. Thus, researchers have no control over the type of data and when they are collected. This difference is also relevant to assessing causal effects. Since an investigator can rarely perform an intervention on the platform, the only way to infer causality is through the exploitation of natural experiments. For example, Coviello et al [14] used adverse weather events to assess emotional contagion in a social network.

Internet services provide a consent mechanism that may include research as one of the purposes of data collection. In practice, people often only implicitly consent to the terms of use through the use of the service, that is, there is a statement indicating that the use of the service implies consent to the terms of use. Although the full terms of service are provided as a link on the service’s page, only a tiny minority of users (fewer than 0.1% in one case) click on this link [13]. Other mechanisms of consent (eg, a pop-up window) have not fared significantly better [15]. Overall, descriptions of “Terms of Use” are necessarily broad because they encompass the entire range of reasons for which data are being collected. This is in contrast to traditional data gathering, where explicit consent is required and obtained and where the consent is for specific data that are required for a particular study.

Research is expected to balance societal benefit with individual beneficence [16]. Basic scientific research in traditional settings does not necessarily achieve individual beneficence. Further, it can be argued that if data are collected when people are interacting with an internet service, individual beneficence is achieved by the fact that individuals receive the service they are seeking. For example, when people interact with a social network, they get the value they were seeking; therefore, all other ethical considerations being equal, additional individual beneficence may not be required.

The privacy of research participants has to be protected. Anonymizing data is one way in which this can be achieved. Additionally, in some cases, anonymous data may eliminate the need for informed consent [17]. Many internet services allow individuals to remain anonymous. Indeed, it has been shown that anonymous services are more reliable [18], which is especially important for medical research. We note that malicious researchers could, in some cases [19], deanonymize users. Thus, some internet data could be considered more beneficial than traditional data in that they are anonymous; however, care should be taken to preserve this anonymity.

Another aspect of privacy pertains to the availability of data to researchers. Companies are, in general, careful to limit access to data they collect both to maintain user privacy and because these data represent a significant asset to the companies. Nevertheless, as abundant scientific literature demonstrates, such access was granted to researchers by companies in the past. Indeed, the COVID-19 pandemic has sped up this process, at least for aggregated data [20].

Finally, internet services are used by a large (and growing) percentage of the world’s population. This means that internet data are more representative of any number of other data sources, which are usually limited in their geographic scope and in the sociodemographic strata from which participants are selected. Even hard-to-reach, disadvantaged populations, such as those of low socioeconomic status, prison inmates, and the homeless, have their own communities on websites such as Reddit. The drawback of this diverse representation is that both researchers and ethics committees find it challenging to assess the benefits of studies that use these populations’ data. Traditionally, one of the roles of institutional review boards was to serve as “public representatives” [21]. With internet data, this is often impossible.

All of these unique characteristics pose new challenges, so they require rethinking in relation to proper ethical behavior tailored to the unique and evolving nature of the research.

## What Is Ethics?

The law is binding on every citizen in every country and demands that individuals refrain from especially unacceptable behavior. Morals dictate appropriate behavior toward one’s fellows, whereas professional ethics mandate a person’s behavior in professional and organizational settings. Proper conduct reflects basic ethical concepts for a profession or organization and position or role, as well the social values of the community and the surrounding society.

Professional ethics constitute the organized concept of the practical ideal of behavior in a professional context. This ideal embraces the system of values and principles that provide the basis for cogent practical decisions concerning the appropriate behavior in the circumstances of the particular human activity that is delineated for a profession [22].

Professional ethics comprise a body of systematic knowledge that includes information garnered from empirical facts and theory based on fundamental studies together with skills developed while trying to solve problems relative to the profession. Professional ethics are continually evolving, becoming ever more sophisticated with every profession having people tasked with improving and advancing the systems. Underlying this process is a local understanding (ie, namely, the ability to explain, grounded in knowledge) of what is done in any given profession, and there is a global understanding regarding its nature. Real understanding of the profession is a problem-solving tool that gives rise to a range of professional mandates and prohibitions [23].

Professional ethics explore and attempt to answer the following questions: “What is the good, the proper, and the right thing to do?” “What is appropriate behavior?” “What are the values that differentiate between the good a person elects to do and the bad from which he or she must refrain and distance him or herself?” and “What are the norms and the rules of conduct according to which one should behave to be ethical?”

Some organizations and professional associations write codes of ethics, which are designed to provide a basis for the honor of the profession: professional humility, recognition of the boundaries of the profession, and recognition of one another’s apparent capabilities. Above and beyond all these, they are meant to delineate appropriate, ethical behavior. The intention is to arrive at a clear understanding of why individuals come together in any organization; the interactions within that organization; and its structure, authority, responsibility, etc. At the same time, there is a special apprehension of the profession, for example, “How does a member of the profession perceive him or herself?” “How does an employee see him or herself?” and “What is the common denominator for all the workers in a country and in the world?”

Ethical requirements must be considered in terms of a wider context as they are among the demands of the social envelope and are fundamental values of the society—democracy, honesty, and moral behavior. Society has various expectations of different professionals, such as the expectation that the policeman and the judge (more so than many others) will be responsible for the defense of democracy and will act honestly according to moral principles. If a lawyer or an accountant is caught in a breach of the law, the public’s attitude toward him or her will be different from when a policeman or judge is found to be in a similar situation.

Thus, whereas codes of ethics generally comprise several basic, very general values, including professionalism, honesty, doing good, observing human rights, loyalty, protecting the honor of the profession, preservation of human life, and social responsibility, they also include fundamental principles, often determined by the professional organization upon its establishment, whether explicitly or implicitly. As professional ethics define the professional identity of the individual and the group, the code of ethics of the police, who deal with citizens, is not the same as that of the army, which engages an enemy from a different country, even though both deal with the security of a country’s citizens.

The rules of ethics serve as a compass that guides a member of the profession in making decisions regarding the various dilemmas that cross his or her path. In general, ethics are not enforced through sanctions stipulated in law but rather by processes and reactions in public, social, professional, and normative actions. Ethics are assimilated through acts of leadership, personal example, education, training, and mentoring.

## Methodology

The methodology of analyzing ethical codes in a comparative manner involves several steps. First, it is important to identify the fundamental text that will serve as the basis for comparison. This may be a specific ethical code or a set of principles that are widely accepted within a particular field or industry.

Next, it is necessary to identify the specific codes or principles that will be compared. This may involve reviewing existing ethical codes or developing a set of principles based on the fundamental text.

Once the codes or principles have been identified, the next step is to analyze them in a systematic manner. This may involve breaking down each code or principle into its component parts and comparing them to one another. It may also involve examining the language and structure of each code or principle to identify any common themes or differences.

Finally, the analysis should aim to draw operative conclusions based on the comparison. This may involve identifying areas of overlap or divergence between the codes or principles and determining the implications of these differences for ethical decision-making. Through this process, it is possible to gain a deeper understanding of the ethical values and principles that guide the behavior of individuals and organizations.

## Current Status of the Codes of Ethics in the Sphere of Internet Data

Codes of ethics as they relate to internet data have been discussed in recent years in both legal and ethical contexts, but only limited aspects of ethics have been addressed, in particular those that are technically easier to define and enforce. Thus, the rules that have been written are concerned primarily with maintaining privacy, as, for example, in the General Data Protection Regulation (replacing the older Article 29 Working Party) [24] and the California Consumer Privacy Act [25]. Focusing as they do on privacy issues derived from the various voice and face recognition capabilities, these codes do not deal with ethical challenges related to the medical world. The regulatory lag in relation to scientific progress and the global nature of scientific activity has, over the years, forced various organizations to engage in self-regulation in the form of ethical codes. A variety of ethical codes for the use of data have been written over the years by different organizations. Notable among them are the Data Ethics Framework by the British government [26]; Digital Analytics Association [27]; International Federation of Pharmaceutical Manufactures and Associations [28]; Good Practice Principles for Data Ethics in the Public Sector by the Organisation for Economic Co-operation and Development [29]; The Five Principles of Data Ethics for Business by the Harvard Business School [30]; The Association of Computing Machinery Code of Ethics [31]; and the code of Association of Internet Researchers [32]. Table 1 offers a comparison among these codes.

The common denominator for all these codes is that they deal with general online data issues; therefore, they usually agree on several core values, even when there are slight changes among them. They count transparency, accountability, fairness, and privacy as core values and, as can be seen in Table 1, add several other values.

Whereas the Organisation for Economic Co-operation and Development document is seemingly a collection of general and nonbinding guidelines rather than a list of key values that should constitute an ethical compass, ACM’s detailed and developed code is a combination of values, rules, and norms, while it specifies key values and basic rules. In any case, none the listed codes deal with the uniqueness of these studies when they are linked to medical research based on an online archive.

The various ethics committees in the medical system have not established proper rules in relation to research based on data from the internet and have effectively placed the responsibility on the internet companies. In effect, internet companies look to the medical sector for guidance, but the medical sector expects internet companies to self-regulate, with the result that medical research based on internet data is carried out without adequate control or a moral compass.

**Table 1:** Comparing the values in the various codes of ethics.

Data ethics framework	Digital analytic association	IFPMA^a^	OECD^b^	Harvard Business School	ACM^c^
Transparency	Transparency	Transparency	—^d^	Transparency	—
Accountability	Accountability	Responsibility and accountability	Accountability	—	—
Fairness	—	Fairness and discrimination	—	—	Fair, honest, and trustworthy
—	Privacy	Autonomy	—	Privacy	Privacy and confidentiality
—	Consumer control	—	—	—	Avoid harm
—	Education	—	—	—	—
—	—	Data quality	—	—	High quality in the processes and products
—	—	Ethics by design	—	—	—
—	—	Responsible data sharing	—	—	—
—	—	—	Integrity	—	—
—	—	—	Trust	—	—
—	—	—	Caution and monitoring	—	—
—	—	—	—	Ownership	—
—	—	—	—	Intention	—
—	—	—	—	Outcomes	Contribute to society and to human well-being
—	—	—	—	—	Professionalism

^a^IFPMA: International Federation of Pharmaceutical Manufactures and Associations.

^b^OECD: Organisation for Economic Co-operation and Development.

^c^ACM: Association for Computing Machinery.

^d^The empty cells show that this particular code of ethics had no reference to a value found in other codes.

## A Proposal for Data Research Guidelines

We therefore propose the following guidelines, inspired by laws of robotics code of ethics by Isaac Asimov. These laws were, as is the nature of laws, absolute and inflexible in their wording. Here, we try to refine them and thereby propose a new line of thinking to consolidate different (and sometimes contradictory) principles. Although these guidelines are based, in part, on known and recognized values, they give new meaning to each of these values.

However, before we delve into these laws and their meanings, it is important to understand the significant values that underlie them, as follows:

### Beneficence

Research should be designed to increase benefit to the people being studied. This is especially important because meaningful consent cannot be obtained. A discussion with the relevant patient groups and caregivers can assist in understanding what would constitute a benefit. Research should balance individual and societal benefits. Therefore, the research should be put in the public domain, and data should be examined as to whether they are suitable for answering the specific research questions.

### Nonmaleficence

Anonymity of individuals should be maintained by both researchers and companies. As far as possible, characteristics pertaining to these individuals or to groups should not be revealed unless relevant to the study and if it does not harm groups in their society. We believe that care has to be taken in characterizing the groups under study that are described by the data to be able to explain to whom the findings refer and not to overfit the data. Therefore, researchers should accurately and transparently describe their findings and not oversell them; further, they should be competent in their fields.

### Justice

As far as possible, researchers should try to address patient communities across the world and in different social, income, and other strata. This is one of the advantages of internet data, and for this reason, such research should be encouraged. Data should be drawn from as many individuals as possible, and researchers should adhere to their professional society’s code of ethics.

### Asimov's Laws for Data Research

In the spirit of the Asimov laws of robotics, one can speak of principled rules of action. We replace the word “robot” with the words “data research” and introduce a few other changes, as follows:

1. First Law: data research should take care to minimize injury to a human being and minimize harm to humans through inaction.
2. Second Law: data research must be regulated by human beings except where such orders would conflict with the First Law.
3. Third Law: data research must be protected if such protection does not conflict with the First or Second Law.
4. Zeroth Law: data research should strive to benefit humanity.

The meaning of these laws for medical research based on internet archive data is first and foremost the understanding that such research can benefit humanity and improve people’s lives. However, as traditional medical research, if performed carelessly, maliciously, or otherwise unprofessionally, it can lead to significant harm. Therefore, researchers should use these data to answer questions pertaining to health and medicine, while realizing that they are dealing with powerful tools that have the potential for misuse.

The unique aspect of Asimov laws is their prioritization of human safety and their incorporation into the programming of robots, ensuring that they always act in a manner that is ethical and beneficial to humanity. The laws serve as a framework for the programming of robots, ensuring that they always act in a manner that is beneficial to humanity and consistent with ethical principles.

These laws are widely recognized as a thought-provoking and influential concept in the field of robotics and artificial intelligence (AI) [33,34]. They highlight the ethical considerations that must be considered when developing and using advanced technologies. The First Law is a crucial principle that ensures the safety of humans in the presence of robots. The Second and Third Laws provide a framework for the responsible use of robots, ensuring that they are used to benefit and serve humanity, rather than causing harm or suffering. Overall, the Asimov laws represent a valuable set of guiding principles for the development and use of robots and AI.

Contrary to the Asimov laws, which assume that the robot may harm humans and therefore it is obliged to obey the human commands and to protect the humans wherever they are, we are now discussing data research in the hands of a human, and it is the human who may harm others because of the information in his or her possession. The responsibility is now in the hands of the owners of the data and whoever conducts the research.

## Discussion and Conclusions

Rapid technological change had led to new and advanced research methods. Much beyond the capabilities of the past, data can now be easily collected and processed. However, technological advancement has brought with it new challenges that have not yet been acknowledged and dealt with by legislators and ethicists around the world. This paper presents the main principles and values in relation to proper behavior when engaging in medical research based on data from the internet, drawing inspiration from the Asimov laws of robotics.

The three Asimov laws, which were first introduced in science fiction, outline a set of ethical guidelines for the use of AI. These laws state that an AI must not harm humans, must follow orders given to it by humans unless those orders conflict with the First Law, and must protect its own existence as long as doing so does not conflict with the first two laws. While these laws have been widely influential in discussions about the ethical use of AI, they have not been widely adopted in practice. In research on AI, the focus is typically on developing and improving the capabilities of the technology, rather than on ensuring that it adheres to a specific set of ethical principles. However, as AI becomes increasingly integrated into various aspects of society, it will be important for researchers and developers to consider the ethical implications of their work and to ensure that AI is used in a responsible and safe manner.

These laws along with some values commonly accepted among members of the relevant professional organizations may help to balance the research and the use of the powerful new tools now at their disposal.

## Abbreviations

AI: artificial intelligence
